# *Miscanthus*: Genetic Diversity and Genotype Identification Using ISSR and RAPD Markers

**DOI:** 10.1007/s12033-014-9770-0

**Published:** 2014-06-01

**Authors:** Sandra Cichorz, Maria Gośka, Anna Litwiniec

**Affiliations:** Research Division in Bydgoszcz, Department of Genetics and Breeding of Root Crops, Plant Breeding and Acclimatization Institute – National Research Institute, Powstańców Wielkopolskich 10, 85-090 Bydgoszcz, Poland

**Keywords:** *Miscanthus*, Genetic diversity, Molecular markers, Identification of ecotypes and varieties, Flow cytometry, Ploidy level

## Abstract

Due to the limited number of molecular studies focused on European gene pool investigation, it is necessary to perform plant material recognition. Eighteen accessions of three *Miscanthus* species, namely, *M.* × *giganteus*, *M. sinensis*, *M. sacchariflorus* were evaluated with the use of molecular marker systems such as: inter simple sequence repeats (ISSRs), random amplified polymorphic DNA (RAPD), and by estimation of ploidy level based on flow cytometry. As a result, only one ISSR primer (ISSR1) and three RAPD primers (RAPD1, RAPD2, RAPD4) were required to identify all genotypes. Moreover, the use of the above mentioned molecular markers enable the proper species recognition of the interspecific hybrid *M.* × *giganteus* “Floridulus,” which has been previously mislabeled as *M. floridulus*. The highest genetic similarity coefficient (0.94) was observed between *M.* × *giganteus* clones, which indicates that the genetic diversity within this species was very low. Whereas *M. sinensis* genotypes represented a relatively wide diversity with similarity coefficient of 0.58. Cluster analysis using UPGMA grouped the 18 accessions in three clusters according to species affiliation including relabeled *M.* × *giganteus* “Floridulus,” which proved to be closely related to *M.*  × *giganteus*. Similar groupings were evident in the PCoA analysis.

## Introduction

Nowadays, due to limited fossil fuels resources and their increasing detrimental effects on the global climate, the biomass production is of particular interest as a renewable source of energy. The main favorable traits of potential bioenergy crop species refer to efficient conversion of free solar power into harvestable biomass with minimal inputs to the environment [1]. Because of a high yield and low environmental requirements, energy grasses such as *Miscanthus* are important crops for biomass production [2]. Vast number of field experiments from distinct regions of Europe demonstrate that *Miscanthus* can achieve the higher energy production in comparison with other energy plants such as annual food crops and woody short rotation coppice species [1, 3, 4] or even distinct perennial grass species [5]. Considering the C4 photosynthesis pathway in these plants, the carbon fixation achieves high rates. The use of nutrients, water so as solar radiation is more efficient in comparison with other plants. All those physiological properties influence adaptation to varied soil and climate conditions. The fact that these grasses are rhizomatous, perennial crops have also a good influence on the lower use of fertilizers required to receive satisfactory biomass yield [6].

The genus *Miscanthus* Anderss. of the *Poaceae* family [7] includes approximately 12 species among which the most valuable species for biomass production are *M. sacchariflorus*, *M. sinensis*, *M.* × *giganteus*, and *M. floridulus* [6]. In Europe, the cultivation of *Miscanthus* is mainly based on *M.* × *giganteus* of tropical and subtropical origin [8, 9]. The *M.* × *giganteus* (2*n* = 3× = 57) is an interspecific hybrid between the diploid *M. sinensis* (2*n* = 2× = 38) and the allotetraploid *M. sacchariflorus* (2*n* = 4× = 76) [10–12]. The efficient biomass productivity of the resulting triploid is caused by a heterosis effect that commonly arises in hybrid cultivars [13]. As a consequence of seed sterility *M.* × *giganteus* is reproduced only vegetatively by rhizome cuttings or in vitro cultures [14, 15], which limits the risk of its release from a cultivation ecosystem to the natural environment [16], but at the same time leads to display very limited genetic diversity. Ideally, there are two or three closely related clones in cultivation [17], but there is a huge probability that European *Miscanthus* wide biomass production is based on one clone [9]. Similar situation is observed in North America, where *M.* × *giganteus* legacy cultivars are expected to be derived via vegetative propagation from a single genet of European origin [18, 19]. Greef et al. [13], using AFLP technique, sampled 31 accessions of *M.* × *giganteus*, 11 clones of *M. sinensis* and two clones of *M. sacchariflorus* that are advisable for cultivation in botanic and market gardens of Middle Europe. From the main *M.* × *giganteus* pool, which indicated low genetic diversity, only three accessions differed, while *M. sinensis* pool showed relatively wide diversity. During similar comparison, Hodkinson et al. [17] also employed AFLP and ISSR markers to characterize genetic resources of 75 accessions from collections at RBG Kew and ADAS Arthur Rickwood Research Station, UK. For the *M.* × *giganteus* accessions (11 taxa), no variation was detected with the use of ISSR markers and little variation most probably due to scoring error with the use of AFLP markers, in contrast to *M. sinensis* accessions (50 taxa) with evident and high level of variation. In another study, De Cesare et al. [20] confirmed that 14 out of 15 *M.* × *giganteus* accessions collected from TCD Botanic Gardens, Dublin, Ireland and University of Hohenheim, Germany that were analyzed with six cpSSR marker loci shared the same haplotype, whereas *M. sinensis* and *M. sacchariflorus* indicated a high level of polymorphism for certain alleles. As mentioned by Ma et al. [21] *M. sinensis* represents highly heterozygous genome. During recent studies performed by the above mentioned research group, with the use of genotyping by sequencing (GBS) the composite linkage map composed of 3,745 SNP markers spanning 2,396 cM on 19 linkage groups was revealed. Moreover, the results indicated that diploid *M. sinensis* is tetraploid origin consisting of two sub-genomes. It showed that sorghum has the closest synthetic relationship to *Miscanthus* in comparison with maize, rice and *Brachypodium distachyon*. Unfortunately, in accordance with literature the designation and distribution of primary particular clones belonging to *M.* × *giganteus* species among either Europe or the USA is dubious and conjectural. The unquestionable fact is that the first *M.* × *giganteus* clone was imported from Japan to Denmark in 1935 by a nursery man, Aksel Olsen as an ornamental plant, and later to North America by commercial clonal propagation [10]. Sacks et al. [22] proposed the above mentioned genotype of *M.* × *giganteus*, which is widespread and predominantly cultivated in Europe and the USA, to be popularly called as “Aksel Olsen,” so as Zub and Brancourt-Hulmel [9]. Taking into consideration the above mentioned facts and the description of *Miscanthus* genotype made by Clifton-Brown and Lewandowski [23] there is a huge probability that “Aksel Olsen” is the spare designation of “Clone Hornum” after the Danish Institute for Landscape Plants at Hornum, where biomass trials with this clone began. According to Sacks et al. [22] later on Deuter and Abraham [24] reported the second clone called *M.* × *giganteus* “Harvey,” which previously existed in Japan and was imported to England about 1980s. Consequently, the origin of different clones could be explained by a distinct natural hybridization event, which occurred in Asia and distributed to Europe. In some collections e.g., The Royal Botanic Garden, Kew and ADAS Arthur Rickwood Research Station *M.* × *giganteus* “Harvey” was incorrectly labeled as *M. sacchariflorus* or *M. sinensis* “Giganteus” and only AFLP analysis revealed the proper taxonomy of this accession [12, 17].

Moreover, in the USA, the most prevalent clone available in the public domain is designed “Illinois” and propagated from a plant growing at the Chicago Botanic Gardens [25], which was originally received from Europe and was of the same genetic identity as the *M.* × *giganteus* genotype widely propagated in Great Britain [26]. This indicated that a narrow gene pool of *M.* × *giganteus* existed. It should be emphasized that cultivation based on genetically uniform unimproved clones is inadequate on the grounds of: disease risk, overwintering problems during the first vegetative season, relatively expensive establishment or varying plant quality requirements for different uses [8, 17, 26, 27]. On account of the above mentioned facts, a crucial factor in *Miscanthus* crop improvement programs is the collection and utilization of diverse germplasms [26].

Unfortunately, little effort has been undertaken to accurately identify cultivars that are available within the germplasm collections of that genus. During the same characterization of a resource collection, Greef et al. [13] indicated, based on AFLP technique, that many of the sampled accessions were inadequately classified as *M. sacchariflorus* instead of *M.* × *giganteus* or *M. sinensis*, whereas Hodkinson et al. [17] accurately assessed 12 cultivars of *Miscanthus* using AFLP accompanied by morphological data. It shows that 16 % of the analyzed accessions were previously unnamed or mislabeled based only on morphological observations.

For the above mentioned reasons, proper choice of the plant material is relevant during cultivation at large areas. As described in a review by Heaton et al. [26] recent efforts of breeding programmes are focused on collection and export of *Miscanthus* germplasms from countries in Southeast Asia. But it requires arrangement of formal partnerships. Furthermore, seeds or propagules must be tested and inspected by a government-approved plant pathologist before release. The current challenge is to screen existing germplasm collections and broaden the genetic base of *M.* × *giganteus* by creating hybrids from wild parents: *M. sinensis* and *M. sacchariflorus* [6]. In view of a relatively high genetic diversity of the parental components as compared with *M.* × *giganteus*, valuable traits could be bred into new varieties [13, 20, 28].

Due to these facts, the estimation of genetic diversity is a prerequisite for the conservation and utilization in breeding programmes [29]. Over the past decade reports in the literature indicate the effective application of molecular markers, based on DNA fingerprinting, used in the studies of *Miscanthus* species. There are potentially many techniques to choose from, such as: RFLP [11], the above mentioned AFLP [12, 13, 17] or SSR [29]. Among these, random amplified polymorphic DNA (RAPD) [30, 31] and inter simple sequence repeats (ISSR) [32] are rapid and inexpensive methods with no requirements of probes or sequence information. They have been widely used in genetic map construction [33] or diversity analysis of *Miscanthus* resources collections held in Europe [17] and the naturally occurring populations [34–36].

Due to the lack of information concerning species identification and characterization of genetic diversity among *Miscanthus* genus available in Poland and limited number of studies focused on the European gene pool, it is necessary to optimize a precise method for plant material recognition. It should be underlined that Poland, so as France and Germany [3], is a promising bioenergy producing region, especially for *Miscanthus*. Moreover, in comparison with Hungary, United Kingdom, Italy and Lithuania in Poland the production, storage and transportation costs are relatively low [2].

The purpose of this research was to genetically evaluate 18 accessions of *Miscanthus* species belonging to the Plant Breeding and Acclimatization Institute – National Research Institute (PBAI – NRI, Research Division in Bydgoszcz, Poland) collection with the use of molecular and cytological observations and select a subset of genotypes that represent the vast majority of diversity within this population. If identified, these accessions could be utilized for breeding and development of *Miscanthus* cultivars. Moreover, we aimed to verify the classification of accessions and define the method for genotype identification. In each accession particular attention was paid to the genetic diversity revealed by ISSR and RAPD molecular markers and ploidy level estimation by flow cytometry. The objectives of this paper focused on three of the *Miscanthus* species studied in Europe for biomass production: *M.* × *giganteus*, *M. sacchariflorus*, *M. sinensis*.

## Materials and Methods

### Plant Material

**Fig. 1:**
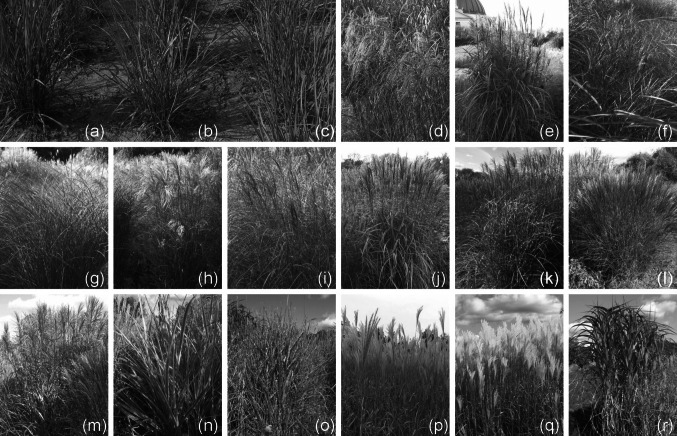
The 18 characterized accessions representing *Miscanthus* species available in the field collection at the PBAI – NRI used in the study. **a** *M.* × *giganteus* “Canada,” **b** *M.* × *giganteus* “Germany,” **c** *M.* × *giganteus* “Great Britain,” **d** *M. sinensis* “Flamingo,” **e** *M. sinensis* “Goliath,” **f** *M. sinensis* “Gracillimus,” **g** *M. sinensis* “Graziella,” **g** *M. sinensis* “Kleine Fontäne,” **h** *M. sinensis* “Kleine Silberspinne,” **i** *M. sinensis* “Malepartus,” **j** *M. sinensis* “Pünktchen,” **k** *M. sinensis* “Rotsilber,” **l** *M. sinensis* “Sirene,” **m** *M. sinensis* “Variegatus,” **o** *M. sinensis* “Zebrinus,” **p** *M. sacchariflorus* ecotype I, **q** *M. sacchariflorus* ecotype II, **r** *M. floridulus* (relabeled as *M.* × *giganteus* “Floridulus”)

In total, 18 accessions representing the *Miscanthus* (Anderss.) species available in the field collection at the PBAI – NRI (Poland) were sampled (Fig. 1a–r). The material included: 12 ornamental varieties of *M. sinensis*, 3 clones (“Canada,” “Germany,” “Great Britain”) of *M.* × *giganteus*, 2 ecotypes of *M. sacchariflorus*, and 1 genotype of *M. floridulus* (relabeled *M.* × *giganteus* “Floridulus”), which are listed in Table 1. The studies were carried out during three vegetative seasons from 2010 to 2012.

**Table 1:** The 18 accessions representing the *Miscanthus* species available in the field collection at the PBAI – NRI used in the study: species, variety/ecotype/clone name, ploidy level

Species	Variety/Ecotype/Clone name	Ploidy level
*M. sinensis*	“Goliath”	3×
*M.* × *giganteus*	“Germany”	3×
*M. floridulus*	Relabeled as *M.* × *giganteus* “Floridulus”	3×
*M.* × *giganteus*	“Great Britain”	3×
*M.* × *giganteus*	“Canada”	3×
*M. sinensis*	“Graziella”	2×
*M. sinensis*	“Kleine Fontäne”	2×
*M. sinensis*	“Flamingo”	2×
*M. sinensis*	“Malepartus”	2×
*M. sinensis*	“Sirene”	2×
*M. sinensis*	“Zebrinus”	2×
*M. sinensis*	“Pünktchen”	2×
*M. sinensis*	“Rotsilber”	2×
*M. sacchariflorus*	Ecotype I	2×
*M. sacchariflorus*	Ecotype II	2×
*M. sinensis*	“Kleine Silberspinne”	2×
*M. sinensis*	“Variegatus”	2×
*M. sinensis*	“Gracillimus”	2×

### DNA Extraction

DNA material was extracted from 1.0 g of fresh leaf material from five plants of each accession, using modified procedure according to Murray and Thompson [37]. The quantity and quality of total genomic DNA were determined by agarose gel electrophoresis and spectrophotometer UV-2100 (Shimadzu, Japan) absorbance at 230, 260, and 280 nm. Only DNA samples with the OD 260/OD 280 > 1.8 and OD 260/OD 230 ≥ 2.0 were diluted in sterile redistilled water and stored at −20 °C until use.

### ISSR PCR

ISSR-PCR reactions were performed in a 25 µL volume of reaction mixture (Thermo Scientific, Fermentas, Germany) containing 25 ng of template DNA, 200 µM of each dNTP, 2.5 mM MgCl_2_, 0.7 U *Taq* polymerase, 1× *Taq* Buffer and 1 µM of each ISSR primer (Genomed, Poland) (Table 2). DNA was amplified in Mastercycler (Eppendorf, Germany) thermocycler and reaction conditions were as followed: 3 min at 94 °C, followed by 40 cycles of 1 min at 94 °C, 1 min at 41–64 °C (depending on the primer sequence), 1 min at 72 °C, and a final extension cycle of 5 min at 72 °C. Thirty-eight primers were tested, out of which 15 generated stable band pattern and were selected for further studies. Amplification products were separated using 1.7 % agarose (Prona, Spain) gel in TBE buffer, stained with ethidium bromide and visualized via GelDoc 2000 UV transilluminator (BioRad, Poland). The reactions were replicated three times with independent DNA extractions to confirm reproducibility of the results.

**Table 2:** List of 15 ISSR and 11 RAPD polymorphic primes, total number of amplified bands, number of polymorphic bands, polymorphism percentage, ISSR primer index, and RAPD primer index, primer sequence used in molecular characterization of 18 *Miscanthus* accessions available in the field collection at the PBAI – NRI

Primer	Total no. of bands	No. of polymorphic bands	Percent polymorphism (%)	ISSR/RAPD primer index	Primer sequence
ISSR					
ISSR1	40	40	100	14.22	(CTG)_7_G
ISSR2	35	35	100	12.67	(GAG)_6_C
ISSR3	34	34	100	12.49	(GAC)_6_T
ISSR4	37	37	100	10.96	(GACA)_5_
ISSR5	36	35	97	10.40	(GTC)_6_A
ISSR6	37	36	97	10.33	(GTG)_6_C
ISSR7	31	30	97	10.02	(CTC)_7_
ISSR8	32	32	100	9.88	(GTG)_6_A
ISSR9	26	26	100	9.07	(AC)_8_TG
ISSR10	31	30	97	8.92	(GAC)_6_
ISSR11	25	23	92	8.03	(GACA)_4_
ISSR12	26	26	100	7.36	(CTC)_7_A
ISSR13	26	25	96	6.95	(GACA)_4_A
ISSR14	14	14	100	4.14	(TC)_8_AG
ISSR15	13	12	92	2.85	TG(TACA)_4_
Mean	30	29	98	9.22	–
Total	443	435	–	–	–
RAPD					
RAPD1	30	29	97	9.23	CCA GCC GAA C
RAPD2	26	26	100	8.46	CCA GCC GAA C
					ATG GAT CCG C
RAPD3	16	16	100	6.02	GTT GCC AGC C
RAPD4	16	15	94	4.77	AGG GAA CGA G
RAPD5	12	12	100	4.67	AGC GCC ATT G
RAPD6	12	10	83	3.80	CCA AGC TGC C
RAPD7	12	10	83	3.35	ACC CGG TCA C
RAPD8	10	8	80	2.87	GGG CTC ATA G
RAPD9	8	6	75	2.49	ATG GAT CCG C
RAPD10	7	7	100	2.28	AGG TGA ACG G
RAPD11	6	6	100	1.80	CGA GTG CCT A
Mean	14	13	94	4.52	–
Total	155	145	**–**	–	–

### RAPD PCR

RAPD-PCR reactions consisted of the following components in 25 µL volume of reaction mixture (Thermo Scientific, Fermentas, Germany): 25 ng template DNA, 200 µM of each dNTP, 2.5 mM MgCl_2_, 0.7 U *Taq* polymerase, 1× *Taq* Buffer, and 1 µM of each RAPD primer (Genomed, Poland) (Table 2). The DNA amplification protocol was: 3 min at 94 °C, followed by 40 cycles of 1 min at 94 °C, 1 min at 29–46 °C (depending on the primer sequence), 1 min at 72 °C, and a final extension cycle of 5 min at 72 °C, conducted in Mastercycler (Eppendorf, Germany) thermocycler. Twenty-six primers were tested, out of which 11 generated stable band patterns and were selected for further studies. Amplification products were electrophoresed in 1.7 % agarose gel (Prona, Spain) and TBE buffer, stained with ethidium bromide and visualized via GelDoc 2000 UV transilluminator (BioRad, Poland). To confirm reproducibility of the results, all of the reactions were repeated three times, with independent DNA extractions.

### Ploidy Level by Flow Cytometry

The young leaf tissue (1 cm^2^) of plants growing in the field was cut off and used for flow cytometry. Samples were prepared according to Galbraith [38] with some modifications. Plant tissue was chopped with razor blade in a Petri dish, containing 2 ml of lysis buffer, with addition of 4′,6-diamidino-2-phenylindole (DAPI) and 2-mercaptoethanol. Suspensions were filtered and the analyses were performed using PAII (Partec, Germany) flow cytometer. For each leaf sample, 5,000–8,000 nuclei were analyzed in five replications, using a logarithmic scale. Histograms were analyzed with the use of a DPAC v.2.2 computer program (Partec Gmbh, Germany).

### Data Analysis

The results of the ISSR-PCR and RAPD-PCR reactions across 18 accessions were processed in a binary system for band presence “1” or absence “0” for each primer. Only reliable, intensive bands were scored. The number of monomorphic and polymorphic amplification products generated by each primer of each marker system was determined. The binary data were used to estimate levels of polymorphism by dividing the polymorphic bands by the total number of bands scored. In agreement with Ghislain et al. [39] the polymorphic index content (PIC) was calculated by the formula: PIC = 1 − *p* ^2^ − *q* ^2^, where *p* is the band frequency and *q* is no-band frequency. So as to show the information content of the ISSR and RAPD primer per assay, the PIC values across alleles for each locus were summed up and named ISSR and RAPD primer index, respectively. Estimates of the genetic similarity were calculated for all accessions according to Nei and Li [40] as follows: *F* = 2*n*_*XY*_/(*n*_*X*_ + *n*_*Y*_), where *n*_*X*_ and *n*_*Y*_ are the numbers of fragments in populations *X* and *Y*, respectively, whereas *n*_*XY*_ is the number of fragments shared by the two populations. Following the terminology of Gower [41] cited by Reif et al. [42], dissimilarity coefficient (*d*) was calculated as: *d* = 1 − *s*, where *s* is the similarity coefficient. According to Gower [43], a dendrogram was constructed using the unweighted pair group method with arithmetic average (UPGMA) [44] and the principal coordinate analysis (PCoA) was performed. The Statistica 7.0 (StatSoft, Poland) software package was used for data management and statistical calculations.

## Results

### ISSR and RAPD Marker Polymorphism

*Miscanthus* species were screened using 15 ISSR primers, which produced reproducible polymorphic banding patterns. A total of 443 bands were scored, of which 435 (98 %) were polymorphic. The number of bands generated per primer varied from 12 to 40. The approximate size of the amplified products ranged from 23 to 3,365 bp. To characterize the capacity of each marker to reveal polymorphic loci among the germplasm, we mainly used the ISSR primer index (Table 2), which revealed that primers: ISSR1, ISSR2, ISSR3 are the most efficient for subsequent fingerprint research in the *Miscanthus* species.

Out of 11 RAPD primers, a total of 155 bands were scored and 145 (94 %) were polymorphic. Amplified DNA fragments varied in size from 138 to 1,613 bp, with 6–29 bands per primer. The RAPD primer index (Table 2) showed that primers: RAPD1, RAPD2, RAPD3, RAPD4 are the most efficient for subsequent fingerprint research in the *Miscanthus* species.

### Genotype and Species-Specific Diagnostic Markers

**Fig. 2:**
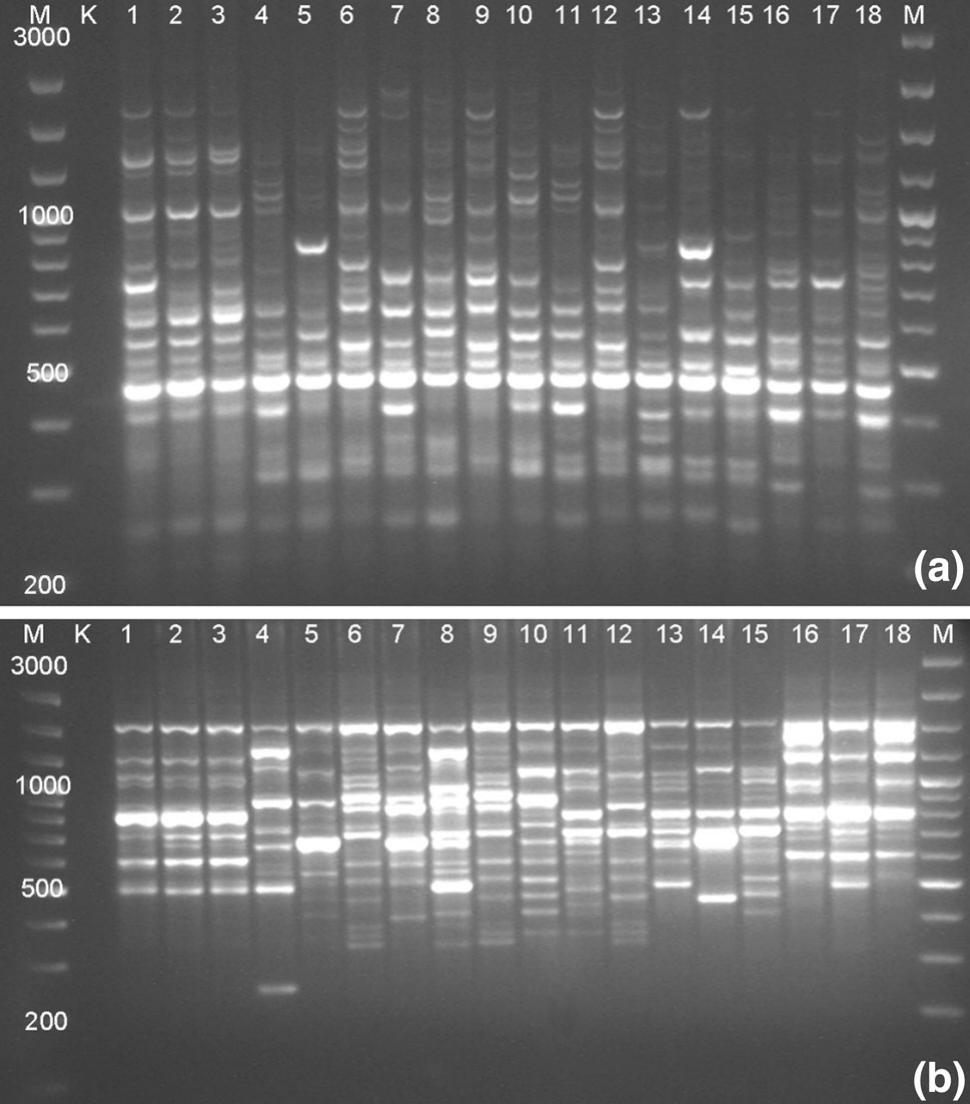
Products of amplification obtained for 18 *Miscanthus* accessions available in the field collection at the PBAI – NRI with the use of selected primers: **a** ISSR1 and **b** RAPD1. Lane M, DNA ladder; K, negative DNA control; 1, *M.* *×* *giganteus* “Canada”; 2, *M.* *×* *giganteus* “Germany”; 3, *M.* *×* *giganteus* “Great Britain”; 4, *M. sinensis* “Flamingo”; 5, *M. sinensis* “Goliath”; 6, *M. sinensis* “Gracillimus”; 7, *M. sinensis* “Graziella”; 8, *M. sinensis* “Kleine Fontäne”; 9, *M. sinensis* “Kleine Silberspinne”; 10, *M. sinensis* “Malepartus”; 11, *M. sinensis* “Pünktchen”; 12, *M. sinensis* “Rotsilber”; 13, *M. sinensis* “Sirene”; 14, *M. sinensis* “Variegatus”; 15, *M. sinensis* “Zebrinus”; 16, *M. sacchariflorus* (ecotype I); 17, *M. sacchariflorus* (ecotype II)*; 18, *M. floridulus* (relabeled as *M.* *×* *giganteus* “Floridulus”)*. *b line number 17 is represented by *M. floridulus* (relabeled as *M.* ×* giganteus* “Floridulus”) and line number 18 by *M. sacchariflorus* (ecotype II)

Both ISSR and RAPD marker systems could successfully distinguish the 18 *Miscanthus* accessions. Only one primer for ISSR (ISSR1) (Fig. 2a) and three primers for RAPD (RAPD1 (Fig. 2b), RAPD2, RAPD4) were needed to identify all genotypes. The first marker technique from the above mentioned revealed 16 unique bands which were genotype-specific in 8 accessions, whereas the second one revealed 64 unique bands in all accessions. Interestingly, we received accession-specific products of amplification for *M.* × *giganteus* genotypes as follows: 2 for “Canada,” 1 for “Germany,” and 2 for “Great Britain.” For *M. floridulus,* we found five accession-specific bands.

In the current study, we searched for amplification products that would be present in every genotype of given species, but absent in others. For *M.* × *giganteus* clones seven (4 ISSR and 3 RAPD), for *M. sinensis* varieties one (ISSR) and for *M. sacchariflorus* ecotypes eight (5 ISSR and 3 RAPD) species-specific bands were recognized.

### Genetic Diversity

The relationships within tested accessions were reflected by the Nei and Li [40] genetic similarity coefficient given in Table 3, which was calculated for combined data set for both molecular marker systems. A low value represented a low degree of genetic similarity, whereas a high value represented a high degree. The highest genetic similarity coefficient (0.94) was observed between *M.* × *giganteus* clones and between *M. sacchariflorus* (0.82) ecotypes, which indicates that the genetic diversity within these accessions was very low or rather low, respectively, whereas *M. sinensis* genotypes represented a relatively wide diversity with similarity coefficient of 0.58. Considerably higher genetic similarity (0.74) was found between *M.* × *giganteus* and *M. floridulus* accessions.

**Table 3:** Average genetic similarity matrix of Nei and Li [40] coefficient based on ISSR and RAPD patterns for the 18 *Miscanthus* accessions available in the field collection at the PBAI – NRI

	1	2	3	4	5	6	7	8	9	10	11	12	13	14	15	16	17	18
1	**–**																	
2	0.9462	**–**																
3	0.9274	0.9326	**–**															
4	0.5547	0.5621	0.5562	**–**														
5	0.5539	0.5447	0.5556	0.6024	**–**													
6	0.5371	0.5441	0.5577	0.5954	0.6053	**–**												
7	0.5977	0.5894	0.5917	0.6164	0.6321	0.7111	**–**											
8	0.5333	0.5287	0.5346	0.6205	0.5833	0.5827	0.6384	**–**										
9	0.5572	0.5566	0.5624	0.5992	0.5835	0.8190	0.6992	0.6133	**–**									
10	0.5720	0.5753	0.5853	0.6342	0.6903	0.6865	0.6802	0.6508	0.6833	**–**								
11	0.5562	0.5597	0.5661	0.6304	0.6143	0.5932	0.6057	0.5890	0.6176	0.6496	**–**							
12	0.5447	0.5519	0.5697	0.5923	0.6157	0.7847	0.7066	0.5915	0.8054	0.6856	0.6551	**–**						
13	0.4652	0.4595	0.4615	0.4951	0.5627	0.5011	0.5628	0.5011	0.5000	0.5513	0.5897	0.5694	**–**					
14	0.5355	0.5510	0.5451	0.5663	0.5566	0.5504	0.5745	0.5672	0.5396	0.6017	0.6045	0.6065	0.5985	**–**				
15	0.5252	0.5285	0.5265	0.5093	0.5455	0.5272	0.5426	0.5229	0.5294	0.5626	0.5863	0.5402	0.5635	0.6698	**–**			
16	0.5508	0.5619	0.5759	0.5000	0.4966	0.4848	0.5104	0.5172	0.5117	0.5580	0.4793	0.4835	0.4465	0.4968	0.4843	**–**		
17	0.5347	0.5378	0.5480	0.5033	0.4587	0.4549	0.4632	0.5000	0.4713	0.5000	0.4513	0.4780	0.4303	0.4781	0.4601	0.8211	**–**	
18	0.7360	0.7287	0.7393	0.5308	0.5422	0.4980	0.5603	0.5378	0.5395	0.5422	0.5494	0.5214	0.4897	0.5362	0.5916	0.6176	0.5892	**–**

1—*M.* × *giganteus* “Canada”; 2—*M.* × *giganteus* “Germany”; 3—*M.* × *giganteus* “Great Britain”; 4—*M. sinensis* “Flamingo”; 5—*M. sinensis* “Goliath”; 6—*M. sinensis* “Gracillimus”; 7—*M. sinensis* “Graziella”; 8—*M. sinensis* “Kleine Fontäne”; 9—*M. sinensis* “Kleine Silberspinne”; 10—*M. sinensis* “Malepartus”; 11—*M. sinensis* “Pünktchen”; 12—*M. sinensis* “Rotsilber”; 13—*M. sinensis* “Sirene”; 14—*M. sinensis* “Variegatus”; 15—*M. sinensis* “Zebrinus”; 16—*M. sacchariflorus* ecotype I; 17—*M. sacchariflorus* ecotype II; 18—*M. floridulus* (relabeled as *M.* × *giganteus* “Floridulus”)

### Cluster Analysis

**Fig. 3:**
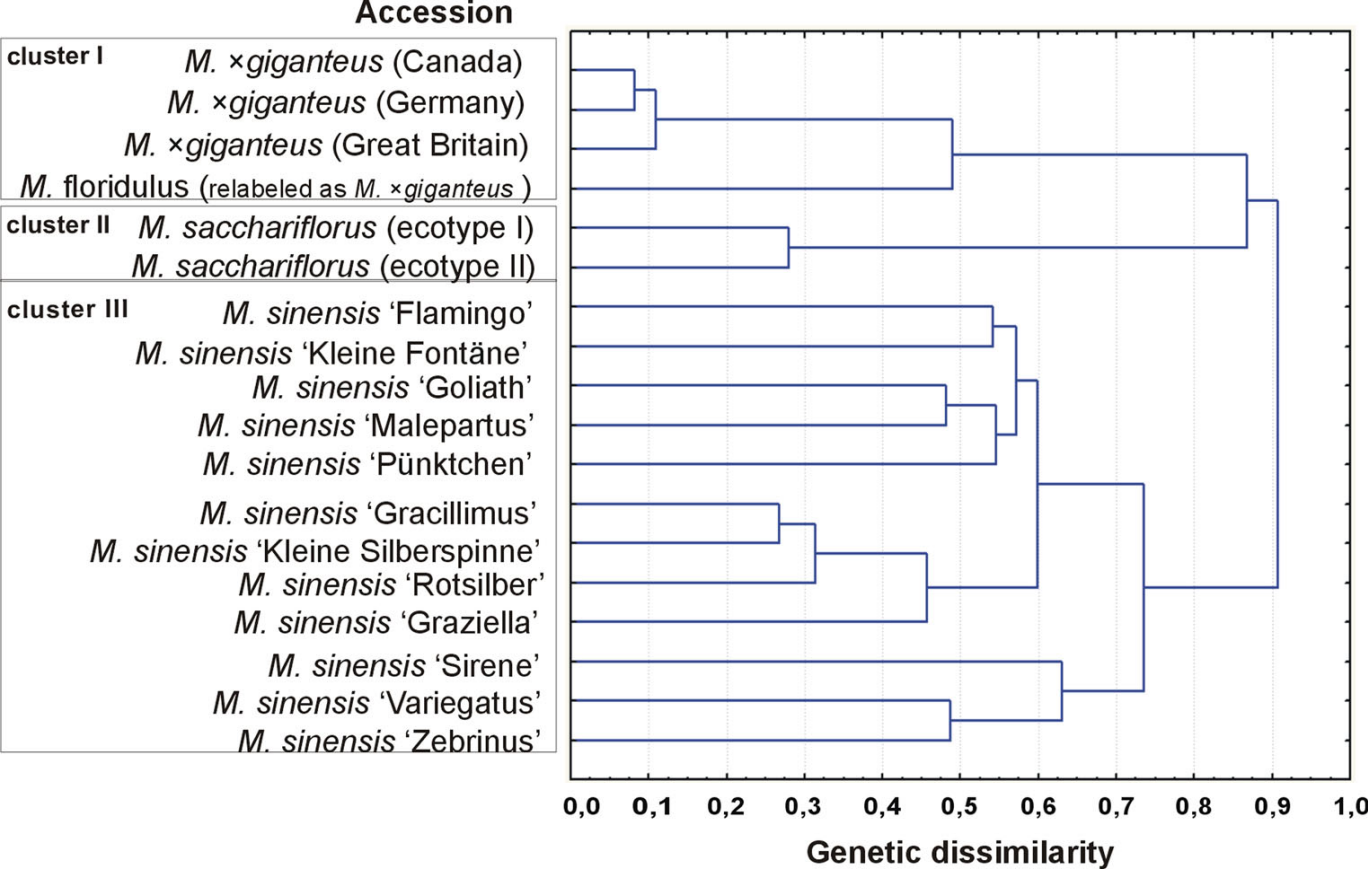
Dendrogram of cluster analysis including 18 *Miscanthus* accessions available in the field collection at the PBAI – NRI constructed from combined ISSR and RAPD data using UPGMA method based on Nei and Li [40] measure of similarity

Cluster analysis based on the matrix of Nei and Li [40] genetic dissimilarity coefficient using UPGMA (Fig. 3) grouped the 18 accessions in three clusters according to species affiliation, apart from *M. floridulus*, which was closely related to *M.* × *giganteus*. The results indicated that the *M.* × *giganteus* “Canada” was more similar to the *M.* × *giganteus* “Germany” than it was to *M.* × *giganteus* “Great Britain,” but the level of variation was very low. The *M.* × *giganteus* “Great Britain” was equidistant from the above mentioned clones. The use of combined ISSR and RAPD markers showed that *M.* × *giganteus* was more closely related to *M. sacchariflorus* than to *M. sinensis*. All the genotypes of *M. sinensis* were grouped together, with the separation on three subclusters. The first one consisted of: “Flamingo,” “Kleine Fontäne,” “Goliath,” “Malepartus” and “Pünktchen”; the second consisted of: “Gracillimus,” “Kleine Silberspinne,” “Rotsilber” and “Graziella”; the third consisted of: “Sirene,” “Variegatus” and “Zebrinus.”

### Principal Coordinate Analysis

**Fig. 4:**
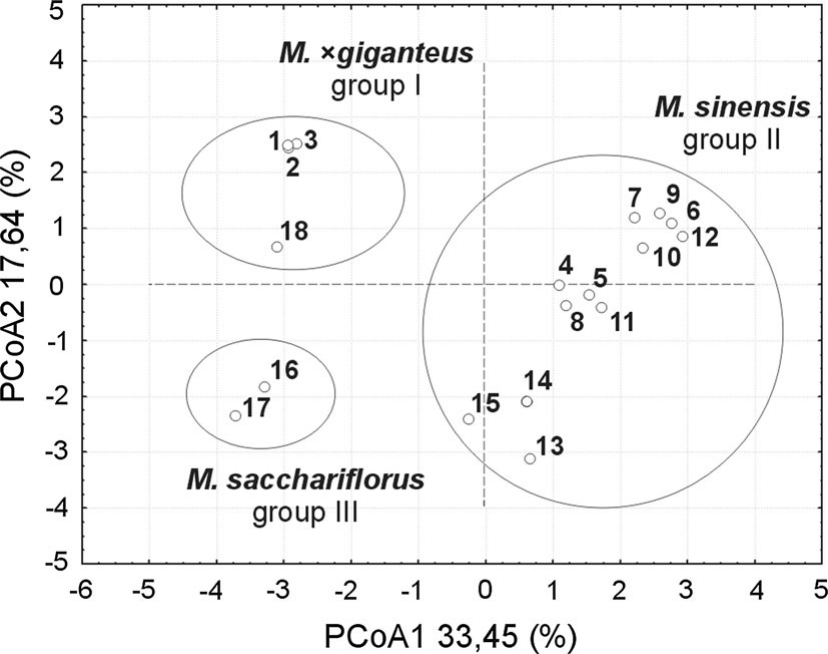
PCoA plot showing the distribution of 18 *Miscanthus* accessions available in the field collection at the PBAI – NRI (Poland) in system of the first two principal coordinates constructed using Nei and Li [40] measure of similarity based on combined ISSR and RAPD data. Numbers given on the chart refer to as follows: 1, *M.* *×* *giganteus* “Canada”; 2, *M.* *×* *giganteus* “Germany”; 3, *M.* *×* *giganteus* “Great Britain”; 4, *M. sinensis* “Flamingo”; 5, *M. sinensis* “Goliath”; 6, *M. sinensis* “Gracillimus”; 7, *M. sinensis* “Graziella”; 8, *M. sinensis* “Kleine Fontäne”; 9, *M. sinensis* “Kleine Silberspinne”; 10, *M. sinensis* “Malepartus”; 11, *M. sinensis* “Pünktchen”; 12, *M. sinensis* “Rotsilber”; 13, *M. sinensis* “Sirene”; 14, *M. sinensis* “Variegatus”; 15, *M. sinensis* “Zebrinus”; 16, *M. sacchariflorus* (ecotype I); 17, *M. sacchariflorus* (ecotype II); 18, *M. floridulus* (relabeled as *M.* *×* *giganteus* “Floridulus”). The accessions can be distinguished using the first two principal coordinates (PCoA1 and PCoA2) and this cumulatively account for 51.09 % (33.45 % and 17.64 %, respectively) of the data variance

Similar groupings were evident in the PCoA analysis (Fig. 4). The first and the second coordinates (designated as PCo1 and PCo2) displayed 33.5 and 17.6 % of the total variation in the combined ISSR and RAPD data. In the first dimension *M. sacchariflorus, M.* × *giganteus and M. floridulus* almost did not differ from each other, whereas the difference was clearly seen, not only between the above mentioned accessions and the genotypes of *M. sinensis*, but also within the latter species. Calculating the second dimension enabled the discrimination between *M. sacchariflorus*, *M.* × *giganteus* and *M. floridulus* accessions. The *M. floridulus* was distinct in its distance to the *M.* × *giganteus* in the second dimension.

### Ploidy Level by Flow Cytometry

**Fig. 5:**
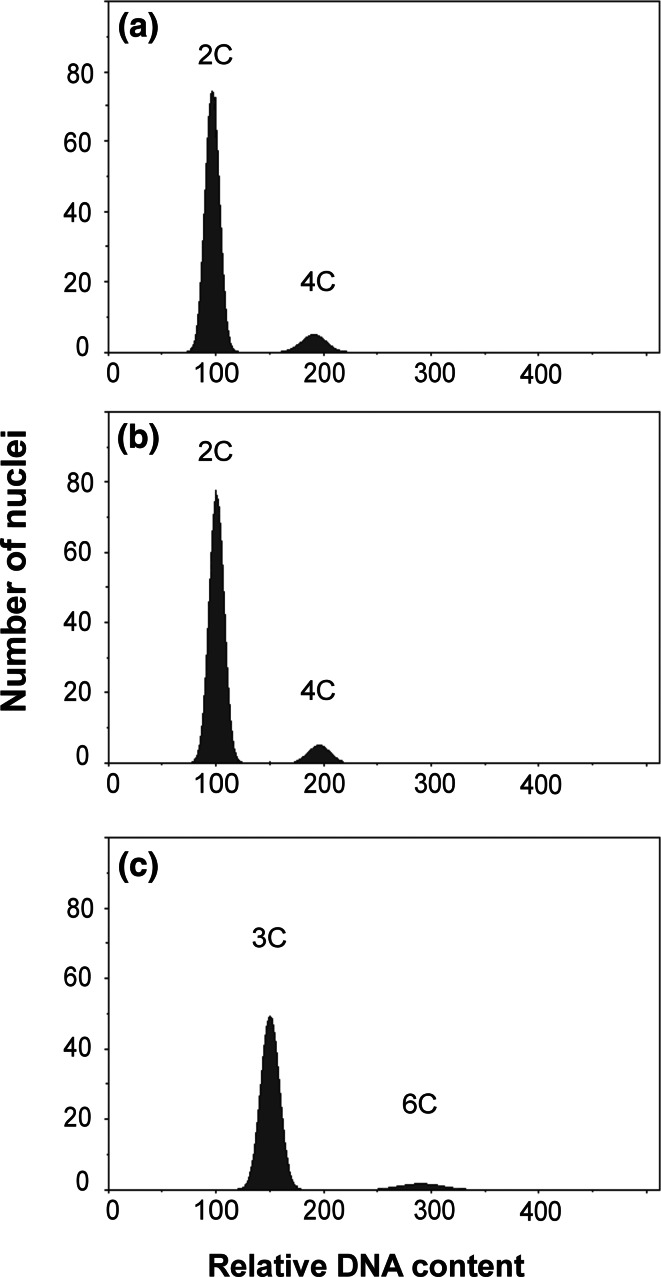
Selected histograms of relative DNA content obtained after analysis of isolated nuclei from *Miscanthus* species. **a** Diploid—a reference *M. sinensis* as a ploidy level control, **b** diploid *M. sinensis* “Flamingo” and **c** triploid *M. sinensis* “Goliath”

The ploidy level of all accessions was estimated using flow cytometry, where a reference diploid *M. sinensis* was used as an external standard (2C and 4C peak adjusted to channels 100 and 200, respectively). Figure 5 shows two types of histograms: diploid-control (Fig. 5a), diploid *M. sinensis* “Flamingo” (Fig. 5b) and triploid *M. sinensis* “Goliath” (Fig. 5c). In our study almost all genotypes from each species did not differ in ploidy level (Table 1). Flow cytometric analyses performed in the same conditions showed that 2C and 4C peaks of *M. sacchariflorus* ecotypes and 11 varieties of *M. sinensis* (apart from “Goliath”) were situated in the same channel as standard, thus were diploids. In contrast, for *M.* × *giganteus* clones, *M. floridulus* and *M. sinensis* “Goliath” 3C peak was found in channel 150 and 6C peak was found in channel 300, which means that those species were triploids.

## Discussion

In *Miscanthus* breeding, the collection and use of diverse germplasms is indispensable. According to Tessier et al. [45] recognition of young plants during multiplication or international exchanges cause many problems in identification of different vegetatively propagated breeding lines. Based only on morphological observations, identification of each species in different vegetative stages, field conditions (environmental variability) or during in vitro propagation is not always sufficient and sometimes even impossible. Hence, an inexpensive, fast and low labor molecular and cytological technique to characterize genotypes and evaluate the genetic diversity is needed.

For that purpose, both ISSR and RAPD marker systems were used in identification of 18 *Miscanthus* accessions. It is worth emphasizing that the comparison of efficiency and utility between ISSR and RAPD markers has not been done in *Miscanthus*, till date. The ISSR method showed slightly higher polymorphism percentage (98 %) as well as wider product size range compared to RAPD marker system (94 %). Considering the fact that the greatest challenge in variety identification is to reduce the number of amplifications and thus the number of primers, which would lower the cost of analysis [45], we recognized the first method as more efficient than the second one. In the current work, ISSR technique revealed higher mean number of polymorphic bands (29) compared to RAPD (13). If it comes to the mean value of marker index, which reveals the information content of primer per assay, likewise it was higher for ISSR (9.22) than RAPD (4.52) markers.

Moreover, the use of only one PCR with the most polymorphic ISSR primer (ISSR1) was enough to distinguish all 18 accessions, whereas RAPD technique required at least three reactions (RAPD1, RAPD2, RAPD4), which would potentially triple the analysis cost. Archak et al. [46] indicated that the cost of ISSR and RAPD analysis per assay of 19 samples was the same for both techniques (USD 80.00), but the cost per polymorphic marker generated was lower in ISSR (USD 7.60) in comparison with RAPD (USD 8.0). To our knowledge, the comparison of these two marker systems has not been done in *Miscanthus* till date, but our observations stay in agreement with results obtained by Esselman et al. [47] who showed higher ISSR than RAPD diversity within four populations of clonally propagated grass *Calamagrostis porter*i ssp. *insperta*. The ISSR method has also been reported to be more useful then RAPD for cultivar identification in numerous plant species, including peanut [48], rice [49], chickpea [50], barley [51], sugarcane [52], and pepper [53]. The distinction between the above mentioned marker systems may concern a different nature of the primer sequence and the amplification of distinct genomic regions. For instance, there is a probability that RAPD bands are associated with functionally important loci, dispersed throughout the genome, whereas ISSR bands are not supposed to be under functional constrains, thus they evolve rapidly and are responsible for higher variability [47]. Moreover, the ISSR markers amplify regions rich in microsatellites, which cause the higher level of polymorphism because of mutations induced by unequal crossing-over and the DNA polymerase slippage during replication [52]. Nevertheless, sometimes only single amplification product of RAPD technique can prove useful in species identification. Kim et al. [34], based on one unique RAPD fragment of *M. sacchariflorus* developed SCAR markers for simultaneous distinction of the *M. sacchariflorus*, *M. sinensis*, and *M.* × *giganteus* species.

In our study, we aimed at finding species-specific markers, which would help in classification of currently retained plant material in field collections and would be useful in verifying the labels of new accessions that can broaden genetic base in the future. For that reason, the use of ISSR technique turned out to be the only possibility in *M. sinensis,* whereas in *M.* × *giganteus* and *M. sacchariflorus* it was more efficient than RAPD marker system. Next step will be to characterize the usefulness of obtained markers in evaluating species identity on larger number of accessions. Moreover, according to Awasthi et al. [54] and Kim et al. [34], further analysis is needed to develop robust species-specific markers so that unique products, after sequencing and designing suitable primers, could be converted to sequence characterized amplification regions (SCARs). Interestingly, despite the presence of five unique accession-specific bands generated by ISSR technique, amplification profiles for *M. floridulus* obtained using this marker system showed very similar banding patterns to *M.* × *giganteus*. Also morphological observations (i.e., inflorescence), nuclear DNA content (data not shown) and ploidy level estimation indicated that this triploid plant, probably incorrectly labeled as *M. floridulus*, should be named as *M.* × *giganteus* “Floridulus.”

At first it seemed that incidental mislabeling appeared during molecular characterizations of *Miscanthus* germplasm collections, which happened as previously mentioned by Greef et al. [13] and Hodkinson et al. [17], but later it turned out that mislabeling of *M. floridulus* is quite frequent. As described in the study by Hodkinson et al. [7, 17], the Neighbor Joining tree of AFLP data revealed that *M. floridulus* (Labill.) Warb. Ex K. Schum. & Lauterb. was grouped with *M. sinensis* accessions and its species status was questionable. Similar results were obtained by Chae [55] for two diploid accessions of *M. floridulus*, which based on the combined morphology, genome size and molecular data, each grouped with *M. sinensis*. The errors in the taxonomic identification between *M. floridulus* and *M. sinensis*, which sometimes cannot be clarified by analytical methods, can be explained by the fact that distribution of these species in the native environment of Pacific region are convergent and thus some intermediates may have appeared [17, 22]. Chouvarine et al. [56] during high-throughput exome sequencing analysis of seven different *Miscanthus* plants aimed at the distinction between closely related genotypes and showed that one plant, named *M. floridulus*, proved to be *M.* × *giganteus*. According to Baldwin [25] misidentified *M. floridulus* indicated the similarity to *M.* × *giganteus*, but also proved to be potential material for development of a new *M.* × *giganteus* cultivar with good morphologic and breeding features of the plant grown under natural conditions (Oktibbeha Country, Mississippi) in comparison with *M.* × *giganteus* “Illinois.” Moreover, Zub et al. [27] performed the identification of key traits for biomass production of 21 clones of four *Miscanthus* species at two harvest dates in Northern France. Results indicated that triploid *M. floridulus* named “*M. floridulus giganteus*” displayed significantly higher canopy and panicle height and shoot diameter than *M.* × *giganteus* clones and the highest value of mean biomass yield (20 t/ha) during second and third crop year in the tested plant group. Authors assumed that it could be hidden cv. *M. floridulus* belonging to the *M.* × *giganteus* species [57].

Because of the above mentioned facts, the overarching objective of our study was to characterize the genetic relationship among all the tested accessions, with the particular attention paid to mislabeled *M. floridulus* genotype and three *M.* × *giganteus* clones. The value of mean dissimilarity coefficient between mislabeled *M. floridulus* and *M.* × *giganteus* clones was higher (0.26) than mean dissimilarity coefficient between *M.* × *giganteus* clones (0.06). The UPGMA analysis, based on Nei and Li [40] measure of similarity showed that all *M.* × *giganteus* genotypes were grouped in one cluster, but were closely related to mislabeled *M. floridulus*, which stayed separately. That was also confirmed in the PCoA analysis. In the first dimension genotypes mentioned above almost did not differ from each other, but the second dimension indicated that mislabeled *M. floridulus* had a distinct distance from *M.* × *giganteus* accessions.

Interestingly, as Baldwin [25] described, such mislabeled genotype of *M. floridulus*, later classified as *M.* × *giganteus*, was a valuable source for the selection of a new cultivar “MSU MFL1” that differed from the “Illinois” clone and other genotypes on the market, giving higher biomass yields.

Moreover, flow cytometry analysis of ploidy level and RAPD molecular markers confirmed the clonal nature of *M.* × *giganteus* genotypes, whereas with the use of ISSR technique we received accession-specific products of amplification for *M.* × *giganteus* genotypes. However, further characterization of upon mentioned bands is needed. Cluster analysis indicated that the *M.* × *giganteus* “Canada” was more similar to the *M.* × *giganteus* “Germany” than it was to *M.* × *giganteus* “Great Britain,” but the level of variation was very low.

In the study made by Głowacka et al. [18] only five accessions of 32 *M.* × *giganteus* legacy cultivars and three of the eight *M.* × *giganteus* polyploids differed for at least one nuclear SSR allele. Authors assumed that genetic diversity within the analyzed group of genotypes from America and Europe nearly did not exist and new crosses would provide genetic variation for this species. According to Greef et al. [13], who evaluated middle European *Miscanthus* species pool with the use of AFLP markers, genetic diversity among 32 accessions of *M.* × *giganteus* was very low and only three accessions could be distinguished from the other. Hodkinson et al. [17] with the use of ISSR markers did not detect genetic variation between *M.* × *giganteus* accessions and that confirmed the clonal nature of analyzed plants. However, the use of AFLP markers enabled detection of a low rate of genetic variation in *M.* × *giganteus* accessions. It allowed to hypothesize that there may be only two or three cultivated clones of *M.* × *giganteus*.

Chouvarine et al. [56] indicated, the availability of multiple genotypes of *M.* × *giganteus* with the use of Illumina high-throughput exome sequencing coupled with SNP mapping and proved that three cultivars studied (“Freedom,” “Illinois,” and “Canada”) are genetically different, which can be exploited in future cultivar development. Unfortunately, the above mentioned studies did not provide technical replications and though it was difficult to verify if the differences are caused by one or more somatic mutations among *M.* × *giganteus* accessions or by sequencing error as it was mentioned by Głowacka et al. [18]. Nevertheless, for introduced populations, which are exposed at novel selection conditions, the genetic differentiation may exist in any ecological trait, which is beneficial [58]. Moreover, in natural environment *Miscanthus* is growing from the subarctic to the subtropics [22]. Nishiwaki et al. [59] founded three different genotypes of *M.* × *giganteus* species, which existed in overlapping populations of *M. sinensis* and *M. sacchariflorus* across Japan and the nucleotide polymorphisms between the sequences of ribosomal DNA internal transcribed spacer (ITS) region were detected. Father investigation in sympatric areas may reveal more natural hybrids between tetraploid *M. sacchariflorus* and diploid *M. sinensis* [60]. On the other hand, serial propagation is a technique that exploits slight somatic mutations occurring in the meristems of vegetatively propagated plants in order to enable selection of individuals with improved cultivation features [24]. This could probably indicate that *M.* × *giganteus* genotypes “Canada,” “Germany” and “Great Britain” characterized in our study belong to one clone origin with the slight differences in genome sequence. However, mislabeled *M. floridulus*, which proved to be *M.* × *giganteus*, may represent the second clone origin, perhaps widespread in Europe and America, which represents another different hybridization event. The above mentioned hypothesis will be verified by further studies with the use of more advanced molecular techniques such as high-throughput exome sequencing or by a wider range of *M. sinensis* and *M. sacchariflorus* accessions as potential maternal components of *M.* × *giganteus*.

On the contrary, we detected that, based on ISSR and RAPD markers, the genetic diversity among *M. sinensis* genotypes was relatively high. That stays in agreement with a number of other studies [13, 17, 29, 36, 61]. Taking into consideration the UPGMA analysis we assigned three subclusters in *M. sinensis* cluster. Those results agreed with PCoA, apart from *M. sinensis* “Malepartus,” which proved higher correlation with accessions from the fourth group of UPGMA analysis. The highest genetic similarity was observed between “Gracillimus” and “Kleine Silberspinne” (0.82). Analyzing the neighbor joining tree of AFLP data of *M. sinensis* accessions revealed by Hodkinson et al. [17], similar grouping between “Goliath” and “Malepartus” can be found. The differences between accessions were quite clearly seen also in genotypes’ phenotypic appearance. Apart from the fact that many ornamental *M. sinensis* cultivars characterize with early flowering and short height, which is undesirable for biomass production [22], a huge phenotypic diversity in a wide range of traits was identified in the UK national collection of *Miscanthus* species among *M. sinensis* genotypes. It is worth emphasizing that those features were connected with yield and quality of plants. The wide geographical distribution may have contributed to that [6] and it can be explained by the different types of reproduction (cross-pollination), ecological habits [36] or as a consequence of breeding selection directed at ornamental values. Moreover, there is a higher probability that genetically differentiated populations better survive distinct environmental conditions [61]. As Farrell et al. [62] indicated *M. sinensis* cultivars are more cold tolerant that *M.* × *giganteus* and, next to the *M. sacchariflorus,* can be used as maternal components for the development of new hybrids [63], for studying of the inheritance of important traits [22] and in comparative genomics for understanding the relationships with other species such as sorghum [21]. In our observations two ecotypes of *M. sacchariflorus* were tested, but they showed rather low genetic diversity and the same ploidy level. As Sacks et al. [22] mentioned *M. sacchariflorus* “Robustus” is planted in living collections or many botanic gardens in Europe and the USA. In order to confirm such origin of *M. sacchariflorus* genotypes analyzed here more studies are needed. It is important to appreciate that the international code of botanic nomenclature defines the hybrid names by its parental components so their ploidy levels should be defined and available [22]. In the above study the ploidy level in *Miscanthus* species ranges from diploid to hexaploid. Basal ploidy in *M. sinensis* is diploid, but natural and artificial polyploids are also common e. g. *M. sinensis* “Goliath” (triploid) [6, 13]. Although *M. sacchariflorus* is normally diploid [22], in this species there is a whole range of ploidy up to hexaploid [6]. As previously mentioned, *M.* × *giganteus* is an interspecific hybrid for which parental components are *M. sinensis* and *M. sacchariflorus* [12] and its ploidy level is typically triploid. However, tetra- and pentaploids, which are produced by artificial hybridizations, may potentially improve biomass cultivars [22]. Our study showed that the need to characterize and broaden the genetic base of *M.* × *giganteus* gene pool still exists not only in Poland, but also in Europe. Moreover, the use of multiple techniques to characterize accessions in field collections is needed. In accordance with previously mentioned studies, it seems that more investigations are required for *M. floridulus* taxonomic recognition as a potential source for genetic improvement of *Miscanthus* species in European conditions [57].
